# Suicide in the Azores Archipelago - a epidemiological study and review

**DOI:** 10.1192/j.eurpsy.2023.2360

**Published:** 2023-07-19

**Authors:** J. Mendes Coelho, M. Bicho, C. Peixoto, H. Fontes

**Affiliations:** Psychiatry, Hospital do Divino Espírito Santo de Ponta Delgada, Ponta Delgada, Portugal

## Abstract

**Introduction:**

The phenomenon of suicide and self-harm is one of the most intriguing and disturbing human behaviours. Suicide is global public health problem, with multiple and complex contributing factors. Global trend show a stabilizing or descending curves in the last years. The Portuguese atlantic archipelago of Azores has had an opposite trend that together with regional proctective and risk factors ought to be addressed for further tailored interventions.

**Objectives:**

Review of the up-to-date literature on this topic and present the recent suicide-related data in the Azores.

**Methods:**

Unsystematic review of the most recent and relevant literature.

**Results:**

Epidemiology, risk and protective factors, preventive and treatment measures were described.

The Azores region has a ascending trend in the suicide mortality rate, opposing the portuguese trend. The azorean suicide mortality rate has a bimodal distribution and has higher values in every age group, except for the +75yo, when compared with the portuguese rates.

**Image:**

**Figure:**
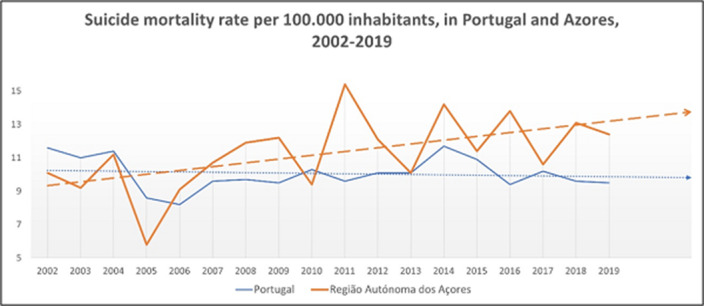

**Image 2:**

**Figure:**
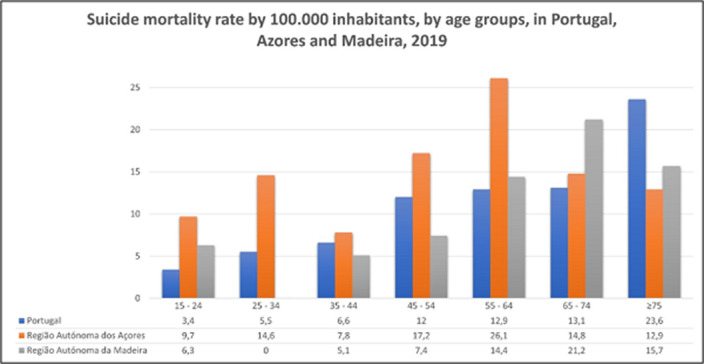

**Conclusions:**

Multiple complex factors contribute to the suicide phenomenon. Suicide protective and risk factors mostly overlap with mental disorders’ factors and those of other health and socio-economic conditions.

Azorean suicide rates are growing against the global and national descending trends.

Prevention and treatment strategies to be implemented regionally must be fine tailored, accounting for the most relevant factors in place, in order to be most effective.

**Disclosure of Interest:**

None Declared

